# Disruptions in serotonin- and kynurenine pathway metabolism in post-COVID: biomarkers and treatment

**DOI:** 10.3389/fneur.2025.1532383

**Published:** 2025-02-13

**Authors:** Carla P. Rus

**Affiliations:** Neuropsychiatrist, Independent Researcher, The Hague, Netherlands

**Keywords:** post-COVID-syndrome (PCS), long COVID, serotonin, tryptophan, 5-hydroxytryptophan (5-HTP), selective serotonin reuptake inhibitors (SSRIs), kynurenine pathway (KP), KP metabolites

## 1 Introduction

This opinion article attempts to connect knowledge about post-COVID syndrome (PCS) gained in neuropsychiatry and immunology. It discusses some misunderstandings about PCS in light of the interplay between the serotonergic system and the kynurenine pathway (KP). From a new perspective, potential biomarkers for further research and therapeutic targets are identified.

Due to the severity and extent of PCS, researchers are urgently searching for its causes and treatments. For neurocognitive and autonomic nervous system problems such as present in PCS, it is common to encounter dysregulated neurotransmitter systems. Among the neurotransmitters, serotonin plays a special role in the immune system and in regulating inflammatory responses by central and peripheral mechanisms (1–5). Serotonin—also known as 5-hydroxytryptamine (5-HT)—is a neurotransmitter with a stimulating effect that influences memory, mood, self-confidence, sleep, emotion, orgasm and eating (6–9).

**Figure 1:**
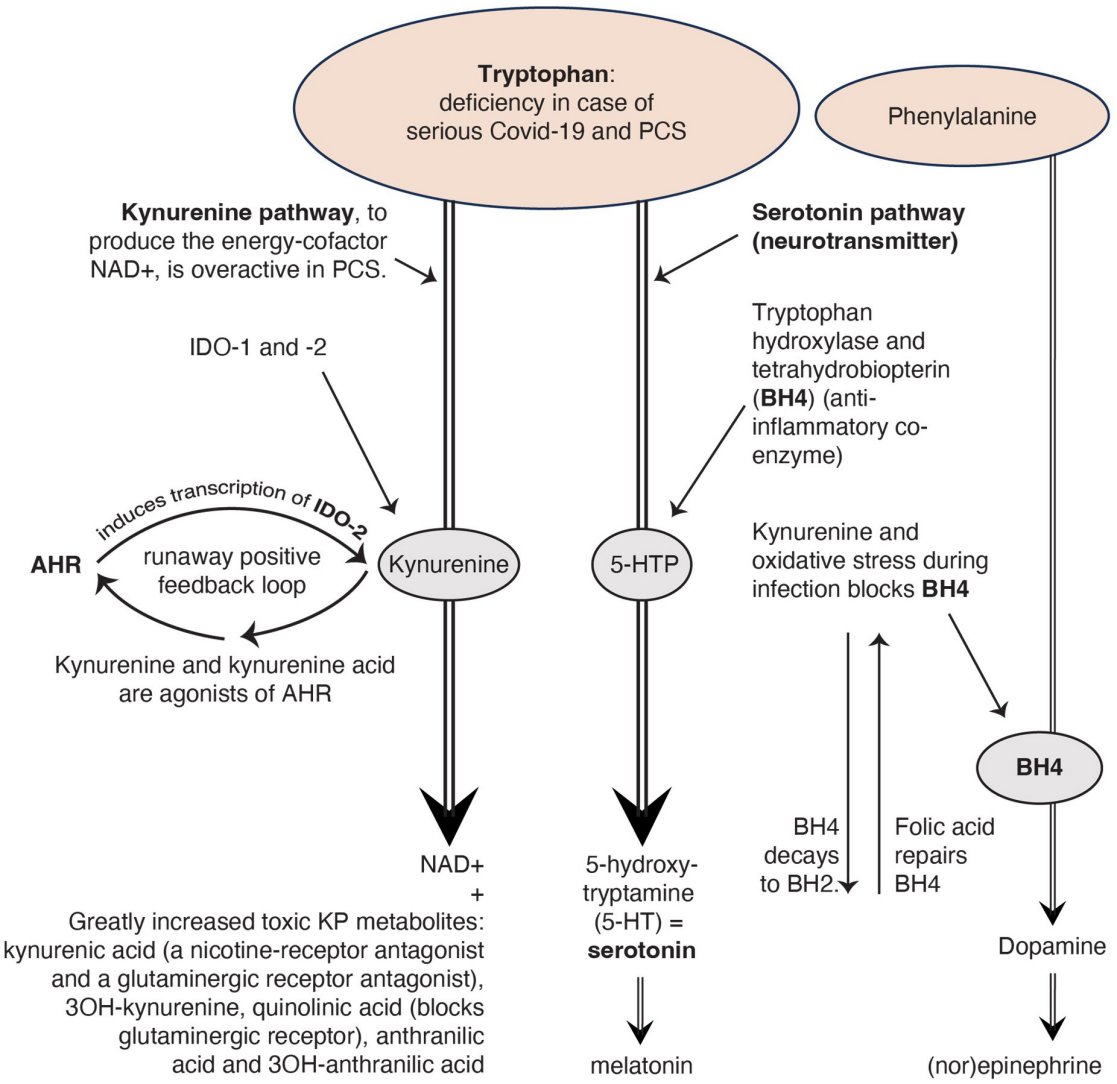
The kynurenine pathway *(KP)* uses the same building block tryptophan as the serotoninergic system. Reproduced from Rus et al. (2023) (13), CC-BY 4.0.

Serotonin not only binds to serotonergic receptors on neurons, but also to receptors on immune cells (3, 5, 10, 11). Many studies indicate that serotonin and its receptors, especially 5-HT3 receptors (one of the serotonin receptors), are involved in the pathogenesis of chronic inflammatory conditions (5, 10, 11). Therapeutic applications of 5-HT3 receptor antagonists for instance have been reported in rheumatoid arthritis (5, 11, 12). An essential amino acid in the serotonin system and also in the KP is tryptophan, a precursor of both serotonin and kynurenine (see Figure 1) and part of a regular diet (14). The KP is a pathway creating an important energy factor and is modulated in conditions as infection and stress (1, 5). Kynurenine regulates the balance between two types of thymus cells (T-cells): regulatory T-cells (Treg-cells), and subsets of T helper 17 cells (Th17 cells) that produce cytokines and have a signaling function (15).

Strong alterations in PCS in intestinal gene expression upregulate genes involved in viral recognition and inflammation pathways and downregulate genes involved in nutrient metabolism, like that of tryptophan (16). This downregulation result in serum serotonin reduction (16). Various researchers suspect this might be the cause of neurocognitive complaints in PCS (13, 16–19).

In this opinion article I address the question whether disruptions in the serotonin- and kynurenine pathway metabolism lead to new biomarkers and treatment in PCS.

## 2 Discussion

### 2.1 Serotonin in five studies: a reliable biomarker in PCS?

In the important study ‘Serotonin reduction in post-acute sequelae of viral infection' by Wong et al. (16) they investigated PCS in four human cohorts, in animal models of viral infection and in organoid cultures. First, they conducted a study among 1,540 PCS patients who presented to a post-COVID center with severe complaints. They identified eight clusters of patients based on clinical symptoms, varying from mainly physical problems, such as loss of strength in muscles, to mainly neurocognitive complaints such as memory disorders. The researchers performed targeted plasma metabolomics on 58 representative PCS patients 3–22 months after infection and found serum serotonin reduction compared with 30 healthy controls.

For this important finding they present three causes: a) diminished intestinal absorption of the serotonin precursor tryptophan. Because of downregulation of genes of the angiotensin converting enzyme (ACE2) these receptors are strongly decreased. Furthermore, not only tryptophan, but also the COVID-19 virus with its spike proteins attaches to these receptors (20, 21). As a consequence, during the COVID-19 infection, tryptophan has to compete with the virus over a reduced number of ACE2 receptors; b) micro-clots of thrombocytes. Thrombocytes contain serotonin. The micro-clots reduce the number of thrombocytes and thus the availability of serotonin; and c) enhanced monoamine oxidase (MAO) that promotes the breakdown of serotonin.

In a study by Sadlier et al. (17), a cohort of 20 hospitalized PCS patients were compared to 20 healthy controls, 4–6 months and 6–9 months after infection. Levels of multiple metabolites with immunomodulatory properties were elevated like quinolinate, a toxic KP metabolite. There were reduced serotonin levels and among other things the serum glutamate (a neurotransmitter) level was increased.

Su et al. (18) performed a longitudinal multi-omic analysis in COVID-19 patients (*n* = 209). This cohort was followed immediately after the COVID-19 infection and had less severe symptoms. They measured autoantibodies, specific COVID-19 RNAemia, metabolic profiles, global plasma proteomic and peripheral blood mononuclear cells (PBMCs) in blood draws. They found no reduced serum serotonin levels compared with 457 healthy controls. What stands out is that the patients reporting neurological symptoms exhibited elevated proteins associated with the negative regulation of the circadian sleep/wake cycle. The hormone melatonin is responsible for this and is produced in the brain (in the pineal gland) from serotonin.

Wong et al. conclude that PCS patients with serious complaints have a greater chance of permanently retaining reduced serotonin levels than PCS patients with mild complaints. They checked this with a cohort of Peluso et al. (22) and found that serum serotonin levels did indeed negatively correlate with the severity of the complaints.

However, in the retrospective study by Mathé et al. (19) no reduced serum serotonin levels were found using the Liquid Chromatography—Mass Spectrometry (lc-ms/ms) technology in a cohort of 34 PCS patients at least 6 months after infection and with serious complaints, which they compared with 14 healthy controls.

Although the study by Wong and colleagues is the most comprehensive of all the studies with interesting and important results, I agree with the conclusion of Mathé and colleagues that serum serotonin is not a reliable biomarker in PCS and should not be used in routine diagnostic assessment, based on two arguments.

### 2.2 Two arguments against serotonin as a biomarker

The first reason is that serotonin cannot cross the blood-brain barrier (14). It appears that only some peripheral serotonin reaches the brain via the cranial nerve, the vagus nerve (16). This nerve normally uses Acetylcholine (Ach) as neurotransmitter (9). So, peripheral serum serotonin level is not directly related to the serotonin level in the brain. Based on animal models, Wong et al. assume that serotonin in the brain is not reduced in PCS. *In vivo*, however, it is technically very difficult to measure serotonin in the brain. With all possible techniques [microdialysis, functional magnetic resonance imaging (fMRI), fast-scan cyclic voltammetry (FSCV), genetically encoded serotonin indicators (GESIs) and positron emission tomography (PET)] either the spatiotemporal resolution is too poor or the technique is too invasive or/and too expensive (23). Wong et al. conclude that with reduced serotonin in the peripheral serum in PCS, less serotonin can move up the vagus nerve to the hippocampus, the control center of memory, possible causing the memory disorders in PCS. In our article in which we describe a study into the treatment of 95 PCS patients with selective serotonin reuptake inhibitors (SSRIs; 16), we give however another explanation. We hypothesize that serotonin reduction also occurs in the brainstem and the brain. After all the pons in the brainstem is the origin of the serotoninergic system and from there, axons are sent throughout the central nervous system (CNS; 6, 7). The afferent vagus nerve also arises from the pons (6, 7) and not from the hippocampus, which Wong and colleagues assume (16). The brainstem is full of ACE2 receptors, to which not only tryptophan but also the COVID-19 virus can attach (20). Hypometabolic areas are found in the pons in PCS (24, 25).

Recent research from Besteher et al. (26) confirms this argument. They found with fMRI scans from PCS patients suffering from neuropsychiatric symptoms (*n* = 30) significantly larger gray matter volumes (GMV) than in healthy controls (*n* = 20). For example in the prefrontal cortex (PFC)—which is involved in a range of higher order cognitive functions and in the hippocampus, control center of memory (27). In these brain areas the neurotransmitter serotonin predominates (27, 28). The authors state the enlargement of the GMV could be a sign of recovery through neurogenesis or compensation (26). Another potential explanation is cerebral swelling caused by immune reactions (26). Given that the neuropsychiatric symptoms persist, it seems likely that the enlargement of the GMV is caused by pathology. Moreover, it provides a plausible explanation for the positive effect of SSRIs on neurocognitive disorders in PCS when there are serotonin balance problems in those brain regions (13).

Furthermore, Su et al. (18) found that melatonin, which is produced in the brain from serotonin, was reduced. This is an additional support—contrary to the conclusion of Wong at al. —that cerebral serotonin may be reduced.

The second reason to reject serotonin as a biomarker, is the variability in the degree of serum serotonin reduction between the cohorts in the different studies (16–19). The causes of this variability can probably be found in the many different variables between the studies. Such as: the time passed between infection and measurement: ranging from 0 to 22 months, the severity of the PCS complaints, their exact quantification (especially for subjective complaints such as neurocognitive complaints) and to which of the eight subgroups the patients belonged in a special cohort. I believe that the methodology used and therefore the results in these studies vary too much to conclude that serotonin is a reliable biomarker in PCS research.

Unlike serotonin, tryptophan can cross the blood-brain barrier (9, 14) and may therefore be a better biomarker option (13, 15). In the case of a comparative study however, the above variables should preferably be more comparable.

### 2.3 Four causes of serotonin reduction

Beside the three causes for the serotonin reduction given by Wong and colleagues, there may be a fourth cause: the KP, a pathway to create the energy factor nicotinamide adenine dinucleotide (NAD+), which interacts extensively with the immune system, seems strongly activated in COVID-19 and PCS (15, 29–31). This results in the formation of many toxic kynurenine metabolites (15, 29–31). This process demands a lot of tryptophan (14; see Figure 1) and because tryptophan is an important precursor of serotonin, a deficiency of tryptophan can also cause a deficiency of serotonin (9).

In the Wong et al. study, the kynurenine metabolites decline as PCS lasted longer. Therefore, the researchers conclude that an activated KP may not be a major cause of serotonin reduction. However, in a study by Guo et al. (30) PCS patients show persistently elevated levels of INDO-2, an enzyme which stimulates the production of kynurenine (Figure 1). In the study by Cron (15) the PCS patients had elevated levels of kynurenine metabolites (such as quinoline), while tryptophan was depleted. Additionally, Cysique et al. found a significant relationship between the level of toxic kynurenine metabolites in blood and the severity of cognitive impairment in PCS (29). These authors conclude that the severity of neurocognitive symptoms seems to be directly related to the degree of overactivity of the KP. The more active the KP, the less tryptophan is left for the production of serotonin.

### 2.4 An overactive KP also causes deficiencies in other hormones and neurotransmitters

Figure 1 illustrates that serotonin deficiency can lead to a melatonin deficiency too. The hormone melatonin regulates the circadian sleep/wake cycle (17, 32). Many PCS patients have sleep problems (13, 33).

Too much kynurenine due to a runaway positive feedback loop of the KP, blocks tetrahydrobiopterin (BH4), a coenzyme for the production of the neurotransmitter dopamine, which in turn ensures the production of the neurotransmitter (nor)epinephrine (9). Norepinephrine from the sympathetic autonomic nervous system increases the frequency and force of muscle contractions (34) why PCS patients with muscle complaints have more symptom reduction with an SNRI (selective serotonin and norepinephrine reuptake inhibitor) compared with an SSRI (13).

If we look at the toxic KP metabolites, we see that both kynurenine acid and quinolinic acid are glutaminergic receptor antagonists. This causes glutamate (a neurotransmitter) accumulation (35) which leads to various problems, such as reduced concentration and palpitations (35), complaints that PCS patients often suffer from (13, 33). That is why we recommend in our article (13) research into N-acetylcysteine as a drug to restore the glutaminergic balance in PCS (35).

### 2.5 Treatment

#### 2.5.1 Tryptophan or 5-HTP?

In one of the experiments of Wong and colleagues (16) they gave tryptophan to mice infected with COVID-19 and suffering from PSC, after which the serotonin levels rose and the mice seemed to recover. In the article “Investigating the Role of Serotonin Levels in Cognitive Impairments Associated with Long COVID-19” of Eslami et al. they advise to treat humans with tryptophan (36). However, tryptophan stimulates—besides the serotoninergic pathway—also the pathological overactive KP and thus the toxic metabolites (15, 29–31). Therefore, I propose that it would be preferable to choose 5-hydroxytryptophan (5-HTP, not to be confused with 5-HT) instead of tryptophan. 5-HTP is a more direct precursor to serotonin that does not feed the KP and that can cross the blood-brain barrier.

#### 2.5.2 SSRIs

An SSRI reduces the reuptake of serotonin and—to a lesser extent—norepinephrine in the presynaptic neuron (9). This allows these additional neurotransmitters in the synapse to transmit their signal to the postsynaptic neuron over a longer period of time (9). SSRIs are usually described for depression or anxiety disorders (37).

Wong and colleagues found that in PCS mice treated with fluoxetine (an SSRI) the cognitive function improved (16). Previously, several researchers found that when patients with COVID-19 took SSRIs, they had a lower chance of developing PCS (38–43).

In our exploratory study we found that two thirds of the PCS-patients showed a considerable or even strong decline of the symptoms after being treated with SSRIs (13). The study by Wong et al. confirmed our hypothesis regarding the importance of the serotoninergic system in PCS. We formulated seven potential mechanisms of action of SSRIs in PCS and one hypothetical mechanism. In short: a. the positive influence of SSRIs on the hypothalamic—pituitary—adrenal-axis [HPA-axis, part of the limbic system; (44–51)], b. the positive influence on the circulatory system (52, 53), c. by prolonging the clotting time which could theoretically help dissolve microclots (54), d. SSRIs lower oxidative stress (52, 53), e. the SSRIs fluvoxamine and fluoxetine have been shown to have extra anti-inflammatory effects by inhibiting sphingomyelinase acid [ASM; (55)], f. SSRIs reduces the pro-inflammatory cytokines interleukin 2 (IL 2) and IL 17 in the CNS (56)—in order to achieve these effects, the SSRI must then be a sigma 1 receptor agonist [an agonist stimulates a receptor; (56)], g. SSRIs also stimulate the production of serotonin cells in the hippocampus (9, 57). Finally, we formulated the hypothesis that SSRIs could slow down the overactive KP (9).

## 3 Conclusion and outlook

Disruptions in the serotonin- and KP metabolism in PCS provide a clear direction for advancing this line of inquiry. While it is evident that many scientists who explore the cause of PCS focus on or the KP route (15, 29–31) or the serotonergic route (16–19, 36), they typically overlook the possibility that these two routes are related.

Additionally, serotonin is not a biomarker to choose for diagnostic assessment of PCS, because it cannot cross the blood-brain barrier (14, 16–19, 22). Tryptophan can cross the blood-brain barrier and may therefore be a better option. In the case of a comparative study however, the variables should preferably be more comparable.

Toxic KP metabolites in serum are good biomarkers as well, because researchers found a significant relationship between the level of toxic KP metabolites in serum and the severity of cognitive impairment in PCS (29).

Various researchers advised to examine the treatment of PCS with an SSRI or with a precursor of serotonin (13, 16, 17, 36). A randomized controlled trial (RCT) on the effect of SSRIs in PCS patients should follow under strict conditions, such as testing the pharmacogenetic profile in advance, since many patients absorb and break down an SSRI too quickly while other patients do this too slowly (13). This can lead to a lack of the desired effect or too many side effects. These patients should be excluded from an RCT with a specific SSRI and can be treated with another SSRI outside the context of the RCT. PCS patients are more sensitive to side effects of SSRIs than other patients (13). Therefore, the trial must also provide for an option to stop increasing the dosage if the balance between effect and side effects threatens to tip without affecting the requirements of an RCT.

Furthermore, a treatment with the precursor tryptophan is not recommended because it also stimulates the overactive KP. Therefore, 5-HTP could be a better option.

This opinion article is also a call for better collaboration between immunologists, neurologists and psychiatrists in the study and treatment of PCS through the field of neuroimmunology. There are already many examples of psychiatric and neurological diseases that are treated immunologically, such as schizophrenia (58–62), childhood depression (61, 63, 64) or multiple sclerosis (65).

There is still much to unravel in neuroimmunology and treatment of immunological disorders with psychotropic drugs should be considered.

## References

[1] Hodo TW de Aquino MTP Shimamoto A Shanker A. Critical neurotransmitters in the neuroimmune network. Front Immunol. (2020) 11:1869. 10.3389/fimmu.2020.01869 32973771 PMC7472989

[2] Attademo L Bernardini F. Are dopamine and serotonin involved in COVID-19 pathophysiology? Eur J Psychiatry. (2021) 35:62–3. 10.1016/j.ejpsy.2020.10.004 33162632 PMC7598536

[3] Wu H Denna TH Storkersen JN Gerriets VA. Beyond a neurotransmitter: the role of serotonin in inflammation and immunity. Pharmacol Res. (2019) 140:100–14. 10.1016/j.phrs.2018.06.015 29953943

[4] Herr N Bode C Duerschmied D. The effects of serotonin in immune cells. Front Cardiovasc Med. (2017) 4:48. 10.3389/fcvm.2017.00048 28775986 PMC5517399

[5] Eteraf-Oskouei T Najafi M. The relationship between the serotonergic system and COVID-19 disease: a review. Heliyon. (2022) 8:e09544. 10.1016/j.heliyon.2022.e09544 35652122 PMC9132783

[6] Salzman C Koester J. “The biology of emotion, motivation, and homeostasis.” In: Kandel E Koester J Mack S Siegelbaum S, editors. Principles of Neural Science. New York: The McGraw-Hill Companies (2021). p. 981–1099.

[7] Kandel E Shadlen M. “Overall perspective.” In:Kandel E Koester J Mack S Siegelbaum S, editors. Principles of Neural Science. New York: The McGraw-Hill Companies (2021). p. 7–127

[8] Shadlen J Kandel E. “Nerve cells, neural circuitry, and behavior.” In: Kandel E Koester F Mack S Siegelbaum S, editors. Principles of Neural Science. New York: The McGraw-Hill Companies (2021). p. 56–73.

[9] Siegelbaum S Fischbach G. “Synaptic transmission.” In:Kandel E Koester J Mack S Siegelbaum S, editors. Principles of Neural Science. New York: McGrawHill (2021). p. 241–385.

[10] Mikulski Z Zasłona Z Cakarova L Hartmann P Wilhelm J Tecott LH. Serotonin activates murine alveolar macrophages through 5-HT [[sb]]2C[[/s]] receptors. Am J Physiol Lung Cell Mol Physiol. (2010) 299:L272–80. 10.1152/ajplung.00032.2010 20495077

[11] Faerber L Drechsler S Ladenburger S Gschaidmeier H Fischer W. The neuronal 5-HT3 receptor network after 20 years of research—evolving concepts in management of pain and inflammation. Eur J Pharmacol. (2007) 560:1–8. 10.1016/j.ejphar.2007.01.028 17316606

[12] Maleki-Dizaji N Eteraf-Oskouei T Fakhrjou A Maljaie SH Garjani A. The effects of 5HT3 receptor antagonist granisetron on inflammatory parameters and angiogenesis in the air-pouch model of inflammation. Int Immunopharmacol. (2010) 10:1010–6. 10.1016/j.intimp.2010.05.013 20646986

[13] Rus CP de Vries BEK de Vries IEJ Nutma I Kooij JJS. Treatment of 95 post-covid patients with SSRIs. Sci Rep. (2023) 13:18599. 10.1038/s41598-023-45072-9 37919310 PMC10622561

[14] Bektas A Erdal H Ulusoy M Uzbay IT. Does seratonin in the intestines make you happy? Turk J Gastroenterol. (2020) 31:721–3. 10.5152/tjg.2020.19554 33169710 PMC7659911

[15] Cron RQ. Immunologic prediction of long COVID. Nat Immunol. (2023) 24:207–8. 10.1038/s41590-022-01396-8 36717724

[16] Wong AC Devason AS Umana IC Cox TO Dohnalová L Litichevskiy L. Serotonin reduction in post-acute sequelae of viral infection. Cell. (2023) 186:4851–67.e20. 10.1016/j.cell.2023.09.013 37848036 PMC11227373

[17] Sadlier C Albrich WC Neogi U Lunjani N Horgan M O'Toole PW. Metabolic rewiring and serotonin depletion in patients with postacute sequelae of COVID-19. Allergy. (2022) 77:1623–5. 10.1111/all.15253 35150456 PMC9111264

[18] Su Y Yuan D Chen DG Ng RH Wang K Choi J. Multiple early factors anticipate post-acute COVID-19 sequelae. Cell. (2022) 185:881–95.e20. 10.1016/j.cell.2022.01.014 35216672 PMC8786632

[19] Mathé P Götz V Stete K Walzer D Hilger H Pfau S. No reduced serum serotonin levels in patients with post-acute sequelae of COVID-19. Infection. (2024). 10.1007/s15010-024-02397-5 39356444 PMC11825522

[20] Sen A. Does serotonin deficiency lead to anosmia, ageusia, dysfunctional chemesthesis and increased severity of illness in COVID-19? Med Hypotheses. (2021) 153:110627. 10.1016/j.mehy.2021.110627 34139598 PMC8180092

[21] Lamers MM Beumer J van der Vaart J Knoops K Puschhof J Breugem TI. SARS-CoV-2 productively infects human gut enterocytes. Science. (2020) 369:50–4. 10.1126/science.abc1669 32358202 PMC7199907

[22] Peluso MJ Kelly JD Lu S Goldberg SA Davidson MC Mathur S. Persistence, magnitude, and patterns of postacute symptoms and quality of life following onset of SARS-CoV-2 infection: cohort description and approaches for measurement. Open Forum Infect Dis. (2022) 9:ofab640. 10.1093/ofid/ofab640 35106317 PMC8755302

[23] Zhao S Piatkevich KD. Techniques for *in vivo* serotonin detection in the brain: state of the art. J Neurochem. (2023) 166:453–80. 10.1111/jnc.15865 37293767

[24] Hugon J Queneau M Sanchez Ortiz M Msika EF Farid K Paquet C. Cognitive decline and brainstem hypometabolism in long COVID: a case series. Brain Behav. (2022) 12:e2513. 10.1002/brb3.2513 35290729 PMC9014998

[25] Ferren M Favède V Decimo D Iampietro M Lieberman NAP Weickert J-L. Hamster organotypic modeling of SARS-CoV-2 lung and brainstem infection. Nat Commun. (2021) 12:5809. 10.1038/s41467-021-26096-z 34608167 PMC8490365

[26] Besteher B Machnik M Troll M Toepffer A Zerekidze A Rocktäschel T. Larger gray matter volumes in neuropsychiatric long-COVID syndrome. Psychiatry Res. (2022) 317:114836. 10.1016/j.psychres.2022.114836 36087363 PMC9444315

[27] Kandel E Siegelbaum E. “Learning, memory, language and cognition.” In: Kandel E Koester J Mack S Siegelbaum S, editors. Principles of Neural Science. New York: The McGraw-Hill Companies (2021). p. 1291–416.

[28] Jarvitch J Sulzer D. “Neurotransmitters.” In:Kandel E Koester J Mack S Siegelbaum S, editors. Principles of Neural Science. New York: The McGraw-Hill Companies (2021). p. 258–378.

[29] Cysique LA Jakabek D Bracken SG Allen-Davidian Y Heng B Chow S. The kynurenine pathway relates to post-acute COVID-19 objective cognitive impairment and PASC. Ann Clin Transl Neurol. (2023) 10:1338–52. 10.1002/acn3.51825 37318955 PMC10424655

[30] Guo L Appelman B Mooij-Kalverda K Houtkooper RH van Weeghel M Vaz FM. Prolonged indoleamine 2,3-dioxygenase-2 activity and associated cellular stress in post-acute sequelae of SARS-CoV-2 infection. EBioMedicine. (2023) 94:104729. 10.1016/j.ebiom.2023.104729 37506544 PMC10406961

[31] Chilosi M Doglioni C Ravaglia C Martignoni G Salvagno GL Pizzolo G. Unbalanced IDO1/IDO2 endothelial expression and skewed keynurenine pathway in the pathogenesis of COVID-19 and post-COVID-19 pneumonia. Biomedicines. (2022) 10:1332. 10.3390/biomedicines10061332 35740354 PMC9220124

[32] Saper CB Elmquist JK. “The brain stem.” In:Kandel ER Koester JD Mack SH Siegelbaum SA, editors. Principles of Neural Sciences. New York: The McGraw-Hill Companies (2020). p. 996–9.

[33] Davis HE McCorkell L Vogel JM Topol EJ. Long COVID: major findings, mechanisms and recommendations. Nat Rev Microbiol. (2023) 21:133–46. 10.1038/s41579-022-00846-2 36639608 PMC9839201

[34] Wolpert D Bastian A. “Movement.” In:Kandel E Koester J Mack S Siegelbaum S, editors. Principles of Neural Science. New York: The McGraw-Hill Companies (2021). p. 713–37.

[35] Rus CP. [A girl with self-harm treated with N-acetylcysteine (NAC)]. Tijdschr Psychiatr. (2017) 59:181–4. 28350146

[36] Eslami Z Joshaghani H. Investigating the role of serotonin levels in cognitive impairments associated with long COVID-19. Chonnam Med J. (2024) 60:141. 10.4068/cmj.2024.60.3.141 39381125 PMC11458317

[37] Sharp T Collins H. Mechanisms of SSRI Therapy and Discontinuation. Curr Top Behav Neurosci. (2023) 66:21–47 10.1007/7854_2023_452 37955823

[38] Bonnet U Juckel G. COVID-19 outcomes: does the use of psychotropic drugs make a difference? Accumulating evidence of a beneficial effect of antidepressants—a scoping review. J Clin Psychopharmacol. (2022) 42:284–92. 10.1097/JCP.0000000000001543 35420565 PMC9042214

[39] Hoertel N Sánchez-Rico M Vernet R Beeker N Jannot A-S Neuraz A. Association between antidepressant use and reduced risk of intubation or death in hospitalized patients with COVID-19: results from an observational study. Mol Psychiatry. (2021) 26:5199–212. 10.1038/s41380-021-01021-4 33536545

[40] Németh ZK Szucs A Vitrai J Juhász D Németh JP Holló A. Fluoxetine use is associated with improved survival of patients with COVID-19 pneumonia : a retrospective case-control study. Ideggyogy Sz. (2021) 74:389–96. 10.18071/isz.74.0389 34856085

[41] Fei L Santarelli G D'anna G Moretti S Mirossi G Patti A. Can selective serotonin reuptake inhibitors/serotonin and norepinephrine reuptake inhibitor antidepressants decrease the “cytokine storm” in the course of COVID-19 pneumonia? Panminerva Med. (2023) 65:321–6. 10.23736/S0031-0808.21.04436-0 34240839

[42] Lenze EJ Mattar C Zorumski CF Stevens A Schweiger J Nicol GE. Fluvoxamine vs. placebo and clinical deterioration in outpatients with symptomatic COVID-19. JAMA. (2020) 324:2292. 10.1001/jama.2020.22760 33180097 PMC7662481

[43] Reis G dos Santos Moreira-Silva EA Silva DCM Thabane L Milagres AC Ferreira TS. Effect of early treatment with fluvoxamine on risk of emergency care and hospitalisation among patients with COVID-19: the together randomised, platform clinical trial. Lancet Glob Health. (2022) 10:e42–51. 10.1016/S2214-109X(21)00448-4 34717820 PMC8550952

[44] Hornig M Gottschalk G Peterson DL Knox KK Schultz AF Eddy ML. Cytokine network analysis of cerebrospinal fluid in myalgic encephalomyelitis/chronic fatigue syndrome. Mol Psychiatry. (2016) 21:261–9. 10.1038/mp.2015.29 25824300

[45] Morris G Anderson G Maes M. Hypothalamic-pituitary-adrenal hypofunction in myalgic encephalomyelitis (ME)/chronic fatigue syndrome (CFS) as a consequence of activated immune-inflammatory and oxidative and nitrosative pathways. Mol Neurobiol. (2017) 54:6806–19. 10.1007/s12035-016-0170-2 27766535

[46] Saper C. The hypothalamus: autonomic, hormonal, and behavioral control of survival. In: Kandel E Koester J Mack S Siegelbaum S, editors. Principles of Neural Science. New York: The McGraw-Hill Company (2021).

[47] Jacobs GE. Pharmacological Aspects of Corticotrophinergic and Vasopressinergic Function Test for HPA Axis Activation. Leiden: Leiden University (2010).

[48] Bao A-M Ruhé HG Gao S-F Swaab DF. Neurotransmitters and neuropeptides in depression. Handb Clin Neurol. (2012) 106:107–36 10.1016/B978-0-444-52002-9.00008-5 22608619

[49] Ruhé HG Khoenkhoen SJ Ottenhof KW Koeter MW Mocking RJT Schene AH. Longitudinal effects of the SSRI paroxetine on salivary cortisol in major depressive disorder. Psychoneuroendocrinology. (2015) 52:261–71. 10.1016/j.psyneuen.2014.10.024 25544738

[50] Bellavance M-A Rivest S. The HPA—immune axis and the immunomodulatory actions of glucocorticoids in the brain. Front Immunol. (2014) 5:136. 10.3389/fimmu.2014.00136 24744759 PMC3978367

[51] Klein J Wood J Jaycox JR Dhodapkar RM Lu P Gehlhausen JR. Distinguishing features of long COVID identified through immune profiling. Nature. (2023) 623:139–48. 10.1038/s41586-023-06651-y 37748514 PMC10620090

[52] Shen W-B Elahi M Logue J Yang P Baracco L Reece EA. SARS-CoV-2 invades cognitive centers of the brain and induces Alzheimer's-like neuropathology. bioRxiv. (2022) 6:2022 10.1101/2022.01.31.478476 35132414 PMC8820661

[53] Hansen R Gaynes B Thieda P Gartlehner G Deveaugh-Geiss A Krebs E. Meta-analysis of major depressive disorder relapse and recurrence with second-generation antidepressants. Psychiatr Serv. (2008) 59:1121–30. 10.1176/appi.ps.59.10.1121 18832497 PMC2840386

[54] Monje M Iwasaki A. The neurobiology of long COVID. Neuron. (2022) 110:3484–96. 10.1016/j.neuron.2022.10.006 36288726 PMC9537254

[55] Niitsu T Iyo M Hashimoto K. Sigma-1 receptor agonists as therapeutic drugs for cognitive impairment in neuropsychiatric diseases. Curr Pharm Des. (2012) 18:875–83. 10.2174/138161212799436476 22288409

[56] Khani E Entezari-Maleki T. Fluvoxamine and long COVID-19; a new role for sigma-1 receptor (S1R) agonists. Mol Psychiatry. (2022) 27:3562–3562. 10.1038/s41380-022-01545-3 35388182 PMC8985056

[57] Shohamy D Schacter D Wagner A. “Learning, memory, language and cognition.” In: Kandel E Koester J Mack SA Siegelbaum S, editors. Principles of Neural Science. New York: The McGraw-Hill Companies (2021). p. 1291–392.

[58] Zandi MS Irani SR Lang B Waters P Jones PB McKenna P. Disease-relevant autoantibodies in first episode schizophrenia. J Neurol. (2011) 258:686–8. 10.1007/s00415-010-5788-9 20972895 PMC3065649

[59] Khandaker GM Cousins L Deakin J Lennox BR Yolken R Jones PB. Inflammation and immunity in schizophrenia: implications for pathophysiology and treatment. Lancet Psychiatry. (2015) 2:258–70. 10.1016/S2215-0366(14)00122-9 26359903 PMC4595998

[60] Pollak TA Lennox BR Müller S Benros ME Prüss H Tebartz van Elst L. Autoimmune psychosis: an international consensus on an approach to the diagnosis and management of psychosis of suspected autoimmune origin. Lancet Psychiatry. (2020) 7:93–108. 10.1016/S2215-0366(19)30290-1 31669058

[61] Dantzer R. Cytokine-induced sickness behaviour: a neuroimmune response to activation of innate immunity. Eur J Pharmacol. (2004) 500:399–411. 10.1016/j.ejphar.2004.07.040 15464048

[62] Zhang J Fu B Wang W Sun C Xu J. Anti-LGI1 antibody-associated encephalitis misdiagnosed as schizophrenia: a case report. Schizophr Bull. (2024) 50:1273–6. 10.1093/schbul/sbae155 39216106 PMC11548918

[63] Khandaker GM Pearson RM Zammit S Lewis G Jones PB. Association of serum interleukin 6 and C-reactive protein in childhood with depression and psychosis in young adult life. JAMA Psychiatry. (2014) 71:1121. 10.1001/jamapsychiatry.2014.1332 25133871 PMC4561502

[64] Ilavská L Morvová M Paduchová Z Muchová J Garaiova I Duračková Z. The kynurenine and serotonin pathway, neopterin and biopterin in depressed children and adolescents: an impact of omega-3 fatty acids, and association with markers related to depressive disorder. A randomized, blinded, prospective study. Front Psychiatry. (2024) 15:1347178. 10.3389/fpsyt.2024.1347178 38414497 PMC10896889

[65] McFarland HF Martin R. Multiple sclerosis: a complicated picture of autoimmunity. Nat Immunol. (2007) 8:913–9. 10.1038/ni1507 17712344

